# Strategies for Coping with Burnout among Palestinian Mental Health Practitioners throughout Wartimes: A Cross-sectional Study

**DOI:** 10.2174/0117450179350988241224114712

**Published:** 2025-01-08

**Authors:** Muna Ahmead, Ahmad. A. Daqqa, Samah Abu Lail, Raba Hadeed, Ikhlass Ghafari

**Affiliations:** 1 Faculty of Public Health, Al Quds University, Jerusalem P.O. Box 51000, Palestine; 2 Palestinian Medical Complex Hospital, Palestinian Ministry of Health, Ramallah, Palestine

**Keywords:** Professional burnout, Coping strategies, Mental health professionals, Psychologists, Political conflict, Palestine

## Abstract

**Background:**

During wartime, mental health professionals are more prone to mental health problems, such as burnout. Currently, there is less knowledge of the coping strategies employed by Palestinian mental health professionals to manage the consequences of wars and conflicts. In light of the ongoing political violence in Palestine, this study sought to investigate the prevalence of burnout and coping methods among mental health professionals.

**Methods:**

The research design was cross-sectional. We used self-reported questionnaires to gather data, including the abbreviated Maslach Burnout Questionnaire and Brief COPE Scale. Multiple regression, Pearson correlation, and a Chi-square test were used to examine the association between the study variables and burn symptoms.

**Results:**

Out of 514 participants, who were surveyed, there was a prevalence of high burnout (75.4%), emotional exhaustion (24.7%), depersonalization (11.9%), and inadequate personal accomplishment (19.6%). Professionals who reported struggling or being unable to interact with their clients during the political violence and those whose psychological state was negatively impacted by it were more likely to feel emotional exhaustion. Also, self-blame was found to raise the chance of emotional exhaustion, whereas planning decreased it. Depersonalization was more common in 31-40-year-olds. Humor and behavioral disengagement increased depersonalization, but religious coping lowered it. Furthermore, treating patients affected by political conflict, being male, not being single, and using substances decreased personal accomplishment, whereas venting increased it.

**Conclusion:**

The results showed an elevated prevalence of burnout among mental health personnel during conflict and political violence. Therefore, it is critical to immediately provide mental health workers with stress management training and psychological support to enhance their psychological well-being. Additionally, they need help in learning how to effectively organize their time, manage activities, and distribute duties during political conflict.

## 1 INTRODUCTION

People in places afflicted by political violence are more likely to experience physical and mental health issues, which can have a substantial influence on their overall well-being [1]. In addition, healthcare services are often interrupted, which leads to a scarcity of both medical supplies and healthcare professionals [2, 3]. It is challenging to provide individualized mental health programs that meet the specific needs of those affected by political conflicts and wars due to a lack of trained mental health professionals and inadequate mental health resources. Experts in the field of mental health include psychologists, psychiatrists, psychiatric social workers, psychiatric nurses, and counselors [4].

Professionals in mental health are primarily drawn to this sector because they have a great desire to help and support people [5]. Mental health practitioners may experience difficulty distinguishing between their identities as individuals and as professionals when confronted with challenges that expressly conflict with the requirements of their work [6]. Furthermore, mental health professionals frequently experience significant levels of stress and burnout, which are exacerbated by the ongoing threat to their safety and the enormous demand for healthcare services [7, 8].

Experiencing excessive exhaustion due to constant stress is known as burnout. It is distinguished by three major characteristics: low energy, emotional alienation from work, and feelings of inefficiency and lack of achievement [9]. Burnout among mental health practitioners is a worldwide problem, with 21% to 67% experiencing high levels [10]. Political violence can create burnout in mental health providers due to demanding working circumstances, anxiety, and devastating events [7, 11]. Furthermore, it is caused by difficult emotional careers, a large workload, long working hours, competing responsibilities, restricted control, impaired autonomy, and unclear goals [12, 13]. According to Leung *et al*. (2022), exposure to a client's traumatic events might cause burnout [14], leaving them unable to deal with daily living [15, 16]. Furthermore, during their regular work, mental health professionals encounter a wide range of emotionally intense situations, including witnessing trauma and grief, as well as the anticipated threat of future harm, aggressive behavior, and the stress of working with traumatized patients [15, 16].

In their study, Cohen *et al*. (2015) revealed that clinicians had the belief that disclosing a traumatic event would lead to adverse outcomes for the therapist. For instance, 20% of the clinicians reported that their client's traumatizing contents elicited upsetting memories from their previous experiences, resulting in emotional disturbance and challenges. Further, 11% of therapists reported experiencing feelings of powerlessness, fear, and vulnerability [17, 18]. Burnout has been associated with increased employee turnover, decreased job engagement, and absenteeism [12]. Burnout can also cause mental health practitioners to have low job satisfaction, somatic disorders, or drug addiction [18]. According to a study, coping mechanisms may increase the likelihood of burnout. There are three main approaches that people use to cope: focusing on the problem, focusing on emotions, or avoiding the problem altogether [19]. Problem-focused coping requires addressing the issue at hand to minimize stress. Emotion-focused coping is the process of managing and regulating emotions that arise in reaction to a distressing occurrence [20]. Avoidance-focused coping is the deliberate act of avoiding or creating distance from a specific problem [21]. Kua *et al*. (2022) reported that problem- and emotion-focused coping approaches are more effective in reducing burnout than avoidance-focused coping [22].

There has been an endless cycle of armed conflict in Palestinian history. Following the war in 1967, the West Bank and Gaza Strip are under continuous occupation by Israeli forces. The protracted conflict in Palestine, between Palestinians and Israelis, has had a profound effect on the Palestinian health care sector, notably since October 7^th^, 2023. The healthcare system has several difficulties, such as inadequate medical resources, declining infrastructure, and restricted healthcare service availability [23]. Moreover, ongoing political instability and frequent incidents of violence have generated a hazardous atmosphere for both patients and mental health professionals [24, 25]. Beyond that, mental health specialists are severely underrepresented in Palestine. For instance, only 38 licensed psychiatrists and 17 licensed psychiatric nurses staff are available at community mental health centers. However, the precise number of counselors, psychotherapists, psychologists, social workers, and counselors is still unknown. The Palestinian Ministry of Health officially accredited and registered 709 mental health practitioners in 2023, according to unreported data [26, 27]. While dealing with victims and vulnerable persons during times of conflict can be incredibly challenging, little is known about the factors that put mental health professionals at risk for mental disorders [28]. In addition, studies focusing on mental health workers in Palestine experiencing burnout due to political conflict are few [29]. This study aimed to determine the prevalence of Palestinian mental health workers’ burnout and how they cope with it in the West Bank. The secondary objective of this study was to determine whether there were associations between burnout and various coping strategies, psychological variables, and demographic factors. Finally, in light of the continuing political violence, the study examined possible factors that contribute to the development of burnout among mental health practitioners.

## 2 METHODS

### 2.1 Study Design and Sampling

STROBE guidelines were used in this study. A descriptive cross-sectional research was carried out between December 22^nd^, 2023, and January 1^st^, 2024, to gather comprehensive data on a certain population at a specific point in time. Anyone practicing mental health in Palestine who is actively involved in treating clients during the ongoing political conflict is a target. This group encompassed university faculty members with expertise in mental health in Palestine, as well as mental health nurses, psychiatrists, social workers, psychologists, and counselors. The exact number of psychologists, social workers, psychotherapists, and counselors in Palestine is unclear. Thus, subjects were selected using snowball and convenience sampling techniques. Due to Israeli military restrictions on movement and closures in Jerusalem and the West Bank, participants were instructed to complete an online questionnaire created with Google Forms. We requested participants to distribute the study link to other mental health practitioners within their network, and we sent them the link through email, social media, messaging application, WhatsApp, and the websites of their professional associations. Five hundred and fourteen participants from the West Bank and Jerusalem completed the questionnaire.

### 2.2 Instruments

The self-report questionnaire of the study consisted of the following three sections:

#### 2.2.1 Section one of the Survey Requested Participants to Fill Out a Socio-demographic Form

Section one of the survey requested participants to fill out a socio-demographic form that included basic personal information, including their age, gender, marital status, occupation, degree of education, place of residence, number of years of experience, and whether or not they had received trauma or crisis training on the job. Participants' perceptions of their inadequacy or inability to interact with their clients were also examined, along with their patient load during the first two months of the current political violence. They were also questioned if they felt their mental health was being impacted negatively by the ongoing political violence. Finally, we inquired as to whether or not they need training in trauma therapy or crisis intervention for victims of political or war-related violence.

#### 2.2.2 The second Section had the Shortened Version of the Maslach Burnout Questionnaire

The second section had the shortened version of the maslach burnout questionnaire [9], which consists of 9 items that measure three subscales: personal accomplishment (PA) (3 items), depersonalization (DP) (3 items), and emotional exhaustion (EE) (3 items). The potential range of the subscale scores is 0–18. A higher degree of burnout is indicated by low scores in personal accomplishment (PA) and high scores in emotional exhaustion (EE) and depersonalization (DP). For EE and DP, a score between 0 and 9 on the subscale was deemed “no to low burnout,” whereas a score between 10 and 18 was deemed “moderate to severe burnout.” On the other hand, when it comes to PA, higher scores indicate less burnout. Questions can be answered using a Likert scale with 7 points, which ranges from 0 (representing “Never”) to 6 (representing “Every day”). The threshold for emotional exhaustion and depersonalization subscales was set at 10 or lower, whereas for personal accomplishment, it was set at 4 or below [9]. The Cronbach's alpha coefficients for emotional exhaustion, depersonalization, and personal accomplishment were 0.82, 0.77, and 0.71, respectively.

#### 2.2.3 The Last Part was the Brief COPE Scale, Developed by Carver [**28**]

The last part was the Brief COPE scale, developed by Carver [30] consisting of twenty-eight items. It included behavioral and cognitive strategies for coping. The respondents score their usage of each approach on a four-point Likert scale, with 1 indicating no use and 4 indicating heavy use. Greater utilization of coping methods is indicated by higher scores. A variety of coping mechanisms are assessed on the Brief COPE scale [28]. Internal consistency is measured by a Cronbach's α value of 0.850. The survey underwent a process of retranslation from Arabic to English following its first translation. Fifteen experts ensured the clarity and correctness of the Arabic language, and twenty mental health professionals participated in the validation process.

### 2.3 Data Analysis

The use of IBM SPSS Statistics (version 25) was employed for data analysis. To do a descriptive analysis, we calculated the percentages and frequencies of the categorical variables and the means and standard deviations of the quantitative variables. Using Pearson correlation and a chi-square test, we examined the relationships between sociodemographic variables, psychological factors, coping mechanisms, and MBI dimensions. Multivariate logistic regression was used to carry out additional analysis on the factors that had statistical significance. To prove statistical significance, a *p*-value lower than 0.05 was considered significant. The study also provided the adjusted odds ratio and its corresponding 95% confidence range.

### 2.4 Ethical Concern and Consent Form

All research procedures adhered to the guidelines laid out in the Declaration of Helsinki. Approval from the Al Quds University Research Ethical Committee was obtained (Ref 347/REC/2023). Anonymity was present in this online survey. At the beginning of the survey, all participants were given written information outlining the goals of the survey and the intended use of the collected data. Participants' responses to the questionnaire served as their agreement to take part in the research.

## 3 RESULTS

This research was carried out with the participation of 514 participants who filled out the survey. Table **1** shows that the majority were women, not single, and aged 40 and above. More than half of the people who took part in the study had bachelor's degrees, and almost the majority were psychologists, counselors, or psychotherapists. Also, the majority of the respondents had been employed in the field of mental health for more than six years. Furthermore, nearly two-thirds of those who took the survey said that their mental health was negatively affected by current political violence. Moreover, over a third of respondents revealed that they had treated victims of the ongoing political violence.

### 3.1 Prevalence of Burnout and its Categories

The overall burnout mean was 11.8 (SD 8.09), with a median of 11 (25^th^ percentile = 6, 75^th^ percentile = 17). Thus, 24.5% had a score less than 6 (n=126), 52.1% had a score between 6-17 (n=268), and 23.3% had a high score more than 17 (n=120). According to the burnout subscale analysis, the prevalence of emotional exhaustion was 24.7% (n=127), 11.9% (n=61) for depersonalization, and 19.6% (n=101) for low personal accomplishment. Additionally, the prevalence of burnout was 24.3% for psychologists, counselors, and psychotherapists, 19.6% for social workers, and 24.0% for psychiatric doctors, psychiatric nurses, and mental health academic staff.

### 3.2 Associations between Respondent Characteristics, Psychological Factors, and Burnout Subscale

Subgroup analysis was conducted to explore the burnout level in different socio-demographics and psychological factors (Table **2**). The findings showed that the emotional exhaustion subscale was significantly associated with participants whose psychological state was negatively affected by current political violence (*p*-value=0.034), participants who reported that they felt disability or inability to deal with patients during current political violence (*p* <0.001), and the participants who need training in crisis intervention and treatment of victims of war and political violence (*p*-value=0.022). Also, depersonalization subscale was associated with participants’ age (*p*-value=0.016), occupation (*p*-value=0.025), and participants who reported that they felt disability or inability to deal with patients during current political violence (*p*-value=0.022). In addition, the personal accomplishment subscale was associated with participants' gender (*p* <0.001), marital status (*p*-value=0.009), and number of patients they treated (*p* <0.001).

### 3.3 Burnout and Coping Strategies

A subgroup analysis was conducted to explore burnout levels in different coping strategies (Table **3**). Emotional exhaustion subscale was significantly associated with active coping (*p*-value=0.028), religion (*p*-value=0.028),), self-blame (*p*-value=0.000), and behavioral disengagement (*p*-value=0.001). Also, depersonalization subscale was significantly associated with humor (*p*-value=<0.001), religion (*p*-value=0.004), self-blame (<*p*-value=0.001), self-distraction (*p*-value=0.008), denial (*p*-value=0.034), substance use (*p*-value=<0.001), and behavioral disengagement (*p*-value=<0.001). In addition, the personal accomplishment subscale was significantly associated with humor (<0.001), self-blame (0.004), acceptance (0.03), religion (0.010), denial (0.013), substance use (<0.001), and behavioral disengagement (0.009).

### 3.4 Multivariate Regression Analysis for Determinants of Burnout Symptom

Multivariate analysis was carried out to investigate variables associated with burnout (Table **4**). Participants who experienced psychological distress due to political violence and used self-blame as a coping strategy were more likely to experience emotional exhaustion, whereas planning decreased it. The 31-40 age group was nearly three times more likely to experience depersonalization than the 18-30 age group. Also, using humor and behavioral disengagement as coping strategies increased depersonalization, but religion decreased it. Several factors, including being male, not being single, treating those suffering from current political violence, and using substances, decreased the likelihood of experiencing personal accomplishment. However, venting increased it.

## 4 DISCUSSION

The findings of our study revealed that over two-thirds of the participants (75.4%) experienced burnout (23.3% displaying a high level of burnout and 52.1% reporting a moderate level of burnout). Furthermore, 24.7% of participants experienced emotional exhaustion, 11.9% had depersonalization, and 19.6% experienced inadequate personal accomplishment. These findings are considered high when compared to other studies in the literature review [31, 32]. Sarma (2018) reported that 32% of psychiatrists in India experienced burnout [33], and Kang *et al*. found that 59.6% of psychological support professionals in Ukraine experienced burnout [34]. Furthermore, Singh *et al*. (2020) demonstrated that 24.2% of the participants encountered burnout [18], whereas Yin *et al*. (2022) reported that 39.9% of mental nurses in China had occupational burnout [35]. Bykov *et al*. found that 25.9% of psychiatrists suffered from burnout, 43.5% had emotional exhaustion, 28.2% exhibited depersonalization, and 32.4% felt low personal accomplishment [36]. Also, in a study conducted by Menaldi *et al*. (2023), it was found that 15.5% of physicians in Indonesia suffered from emotional exhaustion, 5.2% experienced depersonalization, and 39.2% expressed inadequate personal accomplishment [37].

Many factors may account for the elevated prevalence of burnout among mental health professionals in this study. Participants who indicated difficulty or incapacity to handle patients during ongoing political violence, as well as those whose psychological well-being had been negatively affected by it, had a higher likelihood of experiencing emotional exhaustion. This can be because mental health professionals often come into contact with persons who have undergone trauma, such as serious physical injuries or life-threatening situations [38]. Mental health professionals who demonstrate empathy in distressing situations are more prone to experiencing their patients' distress [39]. Therefore, professionals may have difficulties in preserving the patient's empathy, compassion, and warmth during political violence or wars [40]. Consequently, individuals who have experienced burnout in their work lives may exhibit signs of Post-Traumatic Stress Disorder (PTSD). A recent study found that 38.7% of mental health professionals in Palestine reported experiencing PTSD [28]. The current war has seen unprecedented levels of violence compared to previous conflicts or political periods. Palestinian professionals' mental health may suffer because they face the same challenges and political violence as their patients, impacting their view of their own compromised competence. Furthermore, professionals tend to work independently and without supervision under challenging conditions [41] and are unable to seek support owing to confidentiality concerns [42]. Consequently, burnout can be intensified by psychosocial risks, such as difficult clinical settings, exposure to direct trauma, or an excessive workload, all of which can lead to emotional, behavioral, and cognitive disengagement from one's profession and clients [18]. So, to alleviate stress and burnout, mental health professionals in war zones may need quick assistance and psychological treatments. Additionally, they may need groups in which these professionals may express their emotions and provide mutual support.

For emotional exhaustion, the results also indicated that self-blame heightened the probability of experiencing emotional exhaustion, whereas planning decreased it. Other studies have reported similar findings [43-45]. Self-blame, as a negative coping mechanism in the field of medical care, results in heightened levels of stress [45-47]. Excessive reliance on self-blame may indicate professional uncertainty and a deficiency in confidence. Self-blame might result in immobility and impede progress when confronting challenging patient conditions [48]. Spataro *et al*. (2016) reported that self-blame contributes to emotional exhaustion [45]. On the other hand, planning is a strategic method used during the process of evaluating a situation. It aims to explore different cognitive strategies to address the problem [49, 50]. Delaying action-oriented coping techniques might increase stress levels throughout the planning process [51]. Therefore, mental health professionals may alleviate psychological exhaustion by efficiently handling their time, organizing work, sharing responsibilities, and taking measures to minimize exhaustion during instances of political violence.

In addition, for depersonalization, the present study found that depersonalization was three times more prevalent in those aged 31-40 compared to those aged 18-30. According to the findings of a study by Batanda (2024), burnout increases as individuals grow older. Workers who have been employed for a significant period are more prone to burnout as a result of their heightened professional responsibilities. In addition, feelings of frustration or exhaustion when providing treatment might result in less amicable interactions with both clients and coworkers. This might potentially damage the relationship between the patient and the mental health professionals and hinder effective communication [52]. On the other hand, Davies *et al*. (2021) argued that novice counselors and psychotherapists might suffer from 'burnout' as a result of emotional, physical, and mental fatigue. In addition, they may increase their working hours while reducing the amount of time they allocate to interacting with clientele [53]. However, Aguglia *et al*. reported that there was no correlation between age and exhaustion [54]. In addition, the results of the present study indicate that humor and behavioral disengagement were positively associated with depersonalization, but religious coping was negatively associated with depersonalization. Previous studies have yielded comparable findings [44, 45, 55]. Disengagement leads to depersonalization [45], and when individuals participate in behavioral disengagement, they cease their efforts to cope with stress, even when the stress continues [56]. Furthermore, humor plays a beneficial role in enhancing communication, coping mechanisms, relationships, and emotion management in both patients and professionals, thus contributing to their overall well-being [57]. Additional research has indicated that specific forms of humor might either mitigate or exacerbate job stress. For instance, Wang *et al*. (2022) found that burnout is negatively correlated with self-enhancing humor, whereas self-defeating humor is positively correlated with enhancing relationships and reducing stress [58]. Ivancevic *et al*. reported that employing humor as a means of mocking events might manifest cynicism and contribute to burnout [48]. Thus, these tactics are deemed unsuccessful and should be substituted with more efficacious alternatives [48]. Furthermore, our study indicates that good religious coping strategies may assist individuals in adapting to difficult circumstances. In times of stress, individuals may employ religious coping mechanisms to seek psychological comfort and foster personal development [48]. Depersonalization is marked by a lack of emotional connection, intense anger, and a generally negative attitude. Good religious coping techniques can reduce aggressive behavior, indifference, and emotional negativity [55].

Moreover, the current study revealed that personal accomplishment was hindered by treating patients affected by political conflict, being male, and not being single. Other studies have also revealed similar findings [59]. Anbumalar found that males experienced higher levels of distress over career and financial matters, while women exhibited more stress related to family and health concerns [60]. During the conflict in Palestine, the majority of workers were unable to get their salaries. This has resulted in heightened family responsibilities and fatigue [61]. Therefore, professionals must achieve their goals and maintain motivation throughout challenging times by offering incentives to experience a sense of accomplishment. Conversely, Ozumba and Alabere found that men were more involved in personal accomplishment compared to women. There is an expectation for women to prioritize their family above their jobs, which leads to a difference or inequality [32]. Regarding marital status, previous research has indicated that those who are married are more likely to experience burnout compared to those who are single, which aligns with our findings [32, 62]. The burden of family duties can be overwhelming, and entering into marriage might bring about more obligations and lead to emotional and behavioral changes [60, 61]. This might impede their career progression and restrict their achievements. Other studies suggest that marriage might effectively mitigate burnout among professionals by providing enhanced spousal support and stress management [62, 63].

Interestingly, our research revealed that venting enhanced the likelihood of experiencing personal accomplishment, but engaging in substance use diminished it. Venting is the act of expressing strong emotions [64, 65]. Research indicates that those who effectively express their emotions get rapid alleviation of emotional distress. Nevertheless, the inclusion of a supportive “listener” significantly enhances the individual's long-term emotional healing process when they express their emotions [66]. Substance misuse is one example of an avoidant coping strategy that might differ depending on the level of stress [67, 68]. Cecil *et al*. found that excessive alcohol use diminishes the occurrence of burnout syndrome [69]. Substances enhance positive feelings and aid persons in managing negative ones [70, 71, 72]. However, these coping methods diminish stress resilience [73] and offer quick, although transient, alleviation from symptoms [74]. Additionally, substance use has been found to decrease both professional satisfaction and treatment quality [75, 76]. Moreover, individuals in professional occupations who engage in drug addiction have reported a higher number of medical mistakes within the previous three months [75]. Other studies have failed to establish a correlation between substance abuse and burnout [77]. Importantly, the current study used the Brief Cope Scale, which does not inquire about participant's usage of nicotine, alcohol, or opioids. Hence, additional research is necessary to examine the substance employed by mental health professionals in Palestine during instances of political violence.

There are limitations to this study. The ability to demonstrate causal relationships is limited by convenience sampling and cross-sectional methodologies. The use of a self-reported survey also carries the risk of reporting bias. Since Google Docs and WhatsApp were used for recruiting purposes, probably, mental health workers in the Gaza Strip and other impacted areas do not have access to or know how to use these tools. The representativeness of the results may be compromised as a result of this circumstance. Also, there is a lack of a non-mental health worker control group in this study to assess the overall level of burnout and coping in the population. Despite certain restrictions, the results provide light on the mental health of mental health professionals living in countries that experience political violence. As the first study of its kind, this research fills a gap in the literature by investigating mental healthcare workers' experiences of burnout and coping strategies in the context of political and armed violence in Palestine.

### 4.1 Implications for Practice

Thus, professionals should develop healthy coping methods at the outset of their careers to lessen the chances of burnout. Conferences, training programs, or workshops that encourage healthcare workers to consider how their defensive coping mechanisms impact their capacity to manage patients in politically violent situations could be beneficial. Professionals in the mental health field would do well to seek out treatment, psychotherapy, and other forms of psychological assistance to enhance their health, efficiency, and competence on the job. Our findings support the idea that these professionals, particularly those between the age of 31–40, should be screened for this condition at an earlier stage if they exhibit symptoms, such as impaired patient interaction, substance use, negative mental health impacts from political violence, and a tendency toward burnout. To fully understand the causes of mental health professional burnout and effective patient management strategies during political violence, further qualitative and quantitative studies are necessary. To learn which substance abuse Palestinian mental health workers take to cope with the emotional distress brought on by political violence, more studies are required. Finally, additional research incorporating a non-mental health worker control group is necessary for assessing the overall prevalence of burnout and coping strategies among the general population during periods of heightened stress and uncertainty in wars.

## CONCLUSION

Mental health professionals from the Palestinian community experience a high level of burnout during political conflict and war, according to our study. In addition, the likelihood of suffering burnout symptoms is increased when maladaptive coping mechanisms, including self-blame, humor, substance use, and behavioral disengagement, are employed. Psychological intervention and thorough training in stress management are thus urgently needed to help mental health practitioners improve their mental health. They also require guidance in developing skills in time management, prioritization, delegation, and the avoidance of exhaustion caused by political violence and wars.

## Figures and Tables

**Table 1:** Sociodemographic and psychological factors of the participants.

-		N	%
Gender	− Male	165	32.1%
	− Female	349	67.9%
Age	− 18 - 30 years	112	21.8%
	− 31- 40 years	170	33.1%
	− 41+	232	45.1%
Place of residence	− City	241	46.9%
	− Village or refugee camp	273	53.1%
Marital status	− Single	110	21.4%
	− Not single	404	78.6%
Occupation	− Psychologists, counselors, and psychotherapists	368	71.6%
	− Social worker	102	19.8%
	− Psychiatric doctors and nurses and mental health academic staff	44	8.6%
Monthly income	− Less than or equal 1100 $	290	56.4%
	− More than 1100 $	224	43.6%
Education level	− Bachelor degree	309	60.1%
	− Postgraduate degree	205	39.9%
What are your years of experience in the mental health field?	− > 1 year	51	9.9%
	− 1-3 years	81	15.8%
	− 4-6 years	82	16.0%
	− 7-10 years	94	18.3%
	− 11-15 years	82	16.0%
	− 16+ years	124	24.1%
Did you receive any training at work about dealing with the victims of crisis, political violence, and war?	− Yes	384	74.7%
	− No	130	25.3%
Did you receive any training on how to deal with victims of crisis, political violence, and war during your university studies?	− Yes	262	51.0%
	− No	252	49.0%
Did the current political violence have negative effects on your psychological state?	− Yes	460	89.5%
	− No	54	10.5%
Did you treat patients affected by the present political violence in the first two months after it began?	− Yes	221	43.0%
	− No	293	57.0%
How many patients did you treat during the current political violence?	− None	125	24.3%
	− 1-5	148	28.8%
	− 6-15	97	18.9%
	− 16- 45	79	15.4%
	− 46+	65	12.6%
Did you have a feeling of disability or inability to deal with your patients during the current political violence?	− Yes	162	31.5%
	− No	352	68.5%
Do you feel that you need training in crisis intervention and treatment of victims of war and political violence?	− Yes	404	78.6%
	− No	110	21.4%

**Table 2:** Associations between respondent characteristics, psychological history, and burnout subscale.

-		Emotional Exhaustion				-	Personal Accomplishment				-	Depersonalization				-
		<10		≥ 10		-	<10		≥ 10		-	<4		≥ 4		-
		N	%	N	%	*p* value	N	%	N	%	*p* value	N	%	N	%	*p* value
Gender	Male	122	31.5%	43	33.9%	0.625	49	48.5%	116	28.1%	<.001*	139	30.7%	26	42.6%	0.061
	Female	265	68.5%	84	66.1%		52	51.5%	297	71.9%		314	69.3%	35	57.4%	
Occupation	Psychologists, counselors and psychotherapists	270	69.8%	98	77.2%	0.275	68	67.3%	300	72.6%	.218	324	71.5%	44	72.1%	0.025
	Social worker	82	21.2%	20	15.7%		20	19.8%	82	19.9%		95	21.0%	7	11.5%	
	Psychiatric doctors and nurses and mental health academic staff	35	9.0%	9	7.1%		13	12.9%	31	7.5%		34	7.5%	10	16.4%	
Age	18 - 30 years	84	21.7%	28	22.0%	0.356	16	15.8%	96	23.2%	.160	101	22.3%	11	18.0%	0.016*
	31- 40 years	122	31.5%	48	37.8%	-	40	39.6%	130	31.5%	-	140	30.9%	30	49.2%	
	41+	181	46.8%	51	40.2%	-	45	44.6%	187	45.3%	-	212	46.8%	20	32.8%	
Marital status	Single	83	21.4%	27	21.3%	0.964	12	11.9%	98	23.7%	.009*	93	20.5%	17	27.9%	0.190
	Not single	304	78.6%	100	78.7%	-	89	88.1%	315	76.3%	-	360	79.5%	44	72.1%	
Place of residence	City	183	47.3%	58	45.7%	0.751	49	48.5%	192	46.5%	.715	208	45.9%	33	54.1%	0.229
	Village or refugee camp	204	52.7%	69	54.3%	-	52	51.5%	221	53.5%	-	245	54.1%	28	45.9%	
Monthly income	less than or equal 1100 $	218	56.3%	72	56.7%	0.943	56	55.4%	234	56.7%	.826	257	56.7%	33	54.1%	0.697
	More than 1100 $	169	43.7%	55	43.3%	-	45	44.6%	179	43.3%	-	196	43.3%	28	45.9%	
Education level	Bachelor degree	230	59.4%	79	62.2%	0.580	60	59.4%	249	60.3%	.871	276	60.9%	33	54.1%	0.307
	Postgraduate degree	157	40.6%	48	37.8%	-	41	40.6%	164	39.7%	-	177	39.1%	28	45.9%	
What are your years of experience in the mental health field?	> 1 year	38	9.8%	13	10.2%	0.052	12	11.9%	39	9.4%	.747	44	9.7%	7	11.5%	0.069
	1-3 years	64	16.5%	17	13.4%		12	11.9%	69	16.7%		70	15.5%	11	18.0%	
	>3-6 years	58	15.0%	24	18.9%		17	16.8%	65	15.7%		68	15.0%	14	23.0%	
	>6-10 years	64	16.5%	30	23.6%		19	18.8%	75	18.2%		86	19.0%	8	13.1%	
	>10-15 years	58	15.0%	24	18.9%		19	18.8%	63	15.3%		68	15.0%	14	23.0%	
	16+ years	105	27.1%	19	15.0%		22	21.8%	102	24.7%		117	25.8%	7	11.5%	
Did you receive any training at work about dealing with the victims of crisis, political violence, and war?	Yes	292	75.5%	92	72.4%	0.498	69	68.3%	315	76.3%	.099	336	74.2%	48	78.7%	0.446
	No	95	24.5%	35	27.6%		32	31.7%	98	23.7%		117	25.8%	13	21.3%	
Did you receive any training on how to deal with victims of crisis, political violence, and war during your university studies?	Yes	204	52.7%	58	45.7%	0.168	51	50.5%	211	51.1%	.915	226	49.9%	36 25	59.0%	0.181
	No	183	47.3%	69	54.3%		50	49.5%	202	48.9%		227	50.1%		41.0%	
Did the current political violence have negative effects on your psychological state?	Yes	340	87.9%	120	94.5%	0.034*	85	84.2%	375	90.8%	.051	406	89.6%	54	88.5%	0.792
	No	47	12.1%	7	5.5%		16	15.8%	38	9.2%		47	10.4%	7	11.5%	
Did you treat patients affected by the present political violence in the first two months after it began?	Yes	173	44.7%	48	37.8%	0.172	35	34.7%	186	45.0%	.059	196	43.3%	25	41.0%	0.735
	No	214	55.3%	79	62.2%		66	65.3%	227	55.0%		257	56.7%	36	59.0%	
How many patients did you treat during the current political violence?	None	92	23.8%	33	26.0%	0.301	38	37.6%	87	21.1%	<.001*	109	24.1%	16	26.2%	.884
	1-5	108	27.9%	40	31.5%		35	34.7%	113	27.4%	-	128	28.3%	20	32.8%	
	6-15	81	20.9%	16	12.6%		15	14.9%	82	19.9%	-	87	19.2%	10	16.4%	
	16- 45	60	15.5%	19	15.0%		7	6.9%	72	17.4%	-	70	15.5%	9	14.8%	
	46+	46	11.9%	19	15.0%		6	5.9%	59	14.3%	-	59	13.0%	6	9.8%	
Did you have a feeling of disability or inability to deal with your patients during the current political violence?	Yes	104	26.9%	58	45.7%	<0.001*	37	36.6%	125	30.3%	.217	135	29.8%	27	44.3%	0.022*
	No	283	73.1%	69	54.3%		64	63.4%	288	69.7%		318	70.2%	34	55.7%	
Do you feel that you need training in crisis intervention and treatment of victims of war and political violence?	Yes	295	76.2%	109	85.8%	0.022*	83	82.2%	321	77.7%	.328	358	79.0%	46	75.4%	0.518
	No	92	23.8%	18	14.2%		18	17.8%	92	22.3%		95	21.0%	15	24.6%	

**Note: **Significant at *p*-value:0.05.

**Table 3:** Association between coping strategies and burnout subscale.

-		Emotional Exhaustion	Personal Accomplishment	Depersonalization
Active coping	Pearson Correlation	-0.097*	0.082	-0.048
	Sig. (2-tailed)	**0.028**	0.063	0.275
Information support	Pearson Correlation	-0.017	-0.008	0.037
	Sig. (2-tailed)	0.698	0.849	0.405
Positive reframing	Pearson Correlation	-0.008	0.007	0.021
	Sig. (2-tailed)	0.861	0.872	0.637
Planning	Pearson Correlation	-0.035	0.005	0.024
	Sig. (2-tailed)	0.430	0.911	0.594
Emotional support	Pearson Correlation	-0.031	-0.018	0.044
	Sig. (2-tailed)	0.489	0.680	0.318
Venting	Pearson Correlation	0.040	0.072	0.025
	Sig. (2-tailed)	0.369	0.103	0.568
Humor	Pearson Correlation	0.067	-0.176**	0.236**
	Sig. (2-tailed)	0.129	**0.000**	**0.000**
Acceptance	Pearson Correlation	-0.052	0.039	0.018
	Sig. (2-tailed)	0.240	0.375	0.686
Religion	Pearson Correlation	-0.097*	0.114**	-0.192**
	Sig. (2-tailed)	**0.028**	**0.010**	**0.000**
	N	514	514	0.514
Self-blame	Pearson Correlation	0.166**	-0.128**	0.190**
	Sig. (2-tailed)	**0.000**	**0.004**	**0.000**
Self-distraction	Pearson Correlation	0.056	-0.021	0.117**
	Sig. (2-tailed)	0.202	0.638	**0.008**
Denial	Pearson Correlation	0.063	-0.110*	0.093*
	Sig. (2-tailed)	0.154	**0.013**	**0.034**
Substance use	Pearson Correlation	0.027	-0.257**	0.217**
	Sig. (2-tailed)	0.543	**0.000**	**0.000**
Behavioral disengagement	Pearson Correlation	0.152**	-0.115**	0.228**
	Sig. (2-tailed)	**0.001**	**0.009**	**0.000**

**Note: **Significant at *p*-value: 0.05.

**Table 4:** Multivariate regression analysis for determinants of burnout.

-	Emotional Exhaustion					Depersonalization				Personal Accomplishment			
-	Sig.	AOR		95% CI AOR		-	-	95% CI AOR		-	-	95% CI AOR	
				Lower	Upper	Sig.	AOR	Lower	Upper	Sig.	AOR	Lower	Upper
**Age**	-	-	-		-	-	-	-	-	-	-	-	-
− 18 - 30 years	-	-	-		-	-	Ref.	-	-	-	-	-	-
− 31- 40 years	-	-	-		-	**0.012**	2.779	1.251	6.173	-	-	-	-
− 41+	-	-	-		-	0.834	1.092	0.481	2.475	-	-	-	-
**Gender**	-	-	-		-	-	-	-	-	-	-	-	-
− Male	-	-	-		-	-	-	-	-	**0.009**	0.521	0.320	0.848
− Female	-	-	-		-	-	-	-	-	-	Ref.	-	-
**How many patients did you treat during the current political violence?**	-	-	-		-	-	-	-	-	-	-	-	-
− None	-	-	-		-	-	-	-	-	-	Ref.	-	-
− 1-5	-	-	-		-	-	-	-	-	**0.004**	0.248	0.095	0.648
− 6-15	-	-	-		-	-	-	-	-	**0.010**	0.286	0.109	0.745
− 16- 45	-	-	-		-	-	-	-	-	0.132	0.449	0.158	1.273
− 46+	-	-	-		-	-	-	-	-	0.870	0.906	0.277	2.959
Marital status	-	-	-		-	-	-	-	-	-	-	-	-
− Single	-	-	-		-	-	-	-	-	-	Ref.	-	-
− Not single	-	-	-		-	-	-	-	-	**0.031**	0.459	0.226	0.932
**Did the current political violence have negative effects on your psychological state?**	-	-	-		-	-	-	-	-	-	-	-	-
− Yes	**0.042**	2.400	1.031		5.585	-	-	-	-	-	-	-	-
− No	-	Ref.	-		-	-	-	-	-	-	-	-	-
**Did you have a feeling of disability or inability to deal with your patients during the current political violence?**	-	-	-		-	-	-	-	-	-	-	-	-
− Yes	**0.001**	2.084	1.356		3.203	-	-	-	-	-	-	-	-
− No	-	Ref.	-		-	-	-	-	-	-	-	-	-
**Coping Strategies**	-	-	-		-	-	-	-	-	-	-	-	-
− Humor	-	-	-		-	**0.024**	1.258	1.030	1.535	-	-	-	-
− Religion	-	-	-		-	**<.001**	0.684	.576	0.812	-	-	-	-
− Behavioral disengagement	-	-	-		-	**<0.001**	1.485	1.189	1.854	-	-	-	-
− Venting	-	-	-		-	-	-	-	-	**0.041**	1.196	1.007	1.419
− Substance use	-	-	-		-	-	-	-	-	**<0.001**	0.594	0.488	0.723
− Planning	**0.015**	0.821	0.701		0.963	-	-	-	-	-	-	-	-
− Self-blame	**<0.001**	1.321	1.147		1.522	-	-	-	-	-	-	-	-

**Note: **Multivariate logistic regression model: Adjusted for gender, age, living place, monthly income, education level, occupation, years of experience in the mental health field, whether you received any training in your workplace about dealing with the victims of crisis and political violence and war, whether you received any training during your university study about dealing with the victims of crisis, political violence, and war; whether your psychological state was affected negatively during the current political violence, whether you treated patients who suffer from the current political violence, the number of patients they treated, whether you have a feeling of disability or inability to deal with your patients during the current political violence, you feel that you need training in crisis intervention and treatment of victims of war and political violence, active coping, information support, positive reframing, planning, emotional support, venting, humor, acceptance, religion, self-blame, self-distraction, denial, substance use, and behavioral disengagement.

## Data Availability

The data and supportive information are available within the article.

## LIST OF ABBREVIATIONS

PA: = Personal Accomplishment
DP: = Depersonalization
EE: = Emotional Exhaustion

## STANDARDS OF REPORTING

STROBE guidelines were followed.
